# A comprehensive joint analysis of the long and short RNA transcriptomes of human erythrocytes

**DOI:** 10.1186/s12864-015-2156-2

**Published:** 2015-11-16

**Authors:** Jennifer F. Doss, David L. Corcoran, Dereje D. Jima, Marilyn J. Telen, Sandeep S. Dave, Jen-Tsan Chi

**Affiliations:** Department of Molecular Genetics and Microbiology, Duke University, Durham, NC 27710 USA; Center for Genomic and Computational Biology, Duke University, Durham, NC 27708 USA; Department of Medicine, Duke University, Durham, NC 27710 USA; Division of Hematology, Department of Medicine, and Duke Comprehensive Sickle Cell Center, Duke University, Durham, NC 27710 USA

**Keywords:** Erythrocyte, microRNA, Long noncoding RNA, TGF-β

## Abstract

**Background:**

Human erythrocytes are terminally differentiated, anucleate cells long thought to lack RNAs. However, previous studies have shown the persistence of many small-sized RNAs in erythrocytes. To comprehensively define the erythrocyte transcriptome, we used high-throughput sequencing to identify both short (18–24 nt) and long (>200 nt) RNAs in mature erythrocytes.

**Results:**

Analysis of the short RNA transcriptome with miRDeep identified 287 known and 72 putative novel microRNAs. Unexpectedly, we also uncover an extensive repertoire of long erythrocyte RNAs that encode many proteins critical for erythrocyte differentiation and function. Additionally, the erythrocyte long RNA transcriptome is significantly enriched in the erythroid progenitor transcriptome. Joint analysis of both short and long RNAs identified several loci with co-expression of both microRNAs and long RNAs spanning microRNA precursor regions. Within the miR-144/451 locus previously implicated in erythroid development, we observed unique co-expression of several primate-specific noncoding RNAs, including a lncRNA, and miR-4732-5p/-3p. We show that miR-4732-3p targets both SMAD2 and SMAD4, two critical components of the TGF-β pathway implicated in erythropoiesis. Furthermore, miR-4732-3p represses SMAD2/4-dependent TGF-β signaling, thereby promoting cell proliferation during erythroid differentiation.

**Conclusions:**

Our study presents the most extensive profiling of erythrocyte RNAs to date, and describes primate-specific interactions between the key modulator miR-4732-3p and TGF-β signaling during human erythropoiesis.

**Electronic supplementary material:**

The online version of this article (doi:10.1186/s12864-015-2156-2) contains supplementary material, which is available to authorized users.

## Background

Human erythrocytes provide gas transport throughout the body and comprise the majority of cells in whole blood. Human diseases related to red blood cells (RBCs) or erythrocytes, such as anemia or malaria, affect hundreds of millions of people worldwide and present huge health concerns. Although we have gained a significant understanding of how these diseases occur and many treatment options are available, we still cannot explain many aspects of these erythrocyte diseases. Since the precise regulation of both coding and noncoding RNAs is essential for erythrocyte development, these erythrocyte diseases are often accompanied by significant transcriptome changes. During erythropoiesis, microRNAs actively regulate proliferation and/or differentiation of erythroid cells during physiological and pathological adaptions [1]. Dysregulation of various other long and short RNAs also leads to several disease states such as ineffective erythropoiesis and anemias. Circulating erythrocytes can be easily obtained by blood drawing. Accordingly, a detailed analysis of the erythrocyte transcriptome may provide an accessible window into the developmental history and pathophysiology of erythrocytes. However, such an analysis has been deemed impossible since circulating erythrocytes were thought to lack any genetic materials.

During terminal maturation of erythrocytes, the nucleus is extruded from progenitors, leading to anucleate cells with no further RNA production. Therefore, mature erythrocytes were once thought to lack RNAs and have significantly lower signals from RNA-binding dyes such as methylene blue. However, many studies have shown erythrocytes do contain diverse and abundant small RNA species [2], including noncoding RNAs Y1 and Y4 [3] as well as microRNAs [4, 5]. We have previously shown that higher levels of miR-144 and miR-451 reflect the hemolytic phenotype and malaria resistance of sickle erythrocytes, respectively [6, 7]. Additionally, both miR-451 and miR-144 reside in a locus that is regulated by GATA-1, are highly induced during erythroid differentiation, and are critical to erythropoiesis [8]. Therefore, extensive profiling of the RBC transcriptome is critical for an understanding of erythrocyte biology. The RNA composition of erythrocytes may also change during long-term storage for future blood transfusion [9].

However, previous transcriptomic analyses were limited to known erythrocyte microRNAs using microarrays [4], or known microRNAs from mixed reticulocyte (immature red blood cell) and erythrocyte populations using sequencing [10]. In addition, it is not clear whether erythrocytes also contain long (large-sized) RNAs that may provide valuable insights into their development and adaptations. With the recent advances in high-throughput sequencing technologies, it is possible to perform RNA-Seq to identify both known and unknown transcripts.

Here, we employed high-throughput sequencing to characterize both short (small, 18–24 nt) and long (large, > 200 nt) RNAs in human erythrocytes. For long RNA profiling, we prepared RNA-Seq libraries using a protocol that allows for identification of both polyadenylated and non-polyadenylated RNAs. A total of 6843 transcripts were expressed in all three analyzed erythrocyte samples. While this number is far less than that of typical nucleated cells, these analyses established a surprisingly diverse RBC transcriptome. In parallel, short RNA sequencing libraries were prepared and the miRDeep pipeline was utilized to identify both known and putative microRNAs. From these analyses, we identified in mature erythrocytes an abundant, diverse set of microRNAs that include both known and putative microRNAs. The joint analysis of transcriptomes identified several loci with expression of both long and short RNAs, suggesting their coordinated regulation of expression or processing. Furthermore, we performed a functional investigation of the uncharacterized, primate-specific miR-4732-3p within the miR-144/451 locus. MiR-4732-3p was predicted to target both SMAD2 and SMAD4 [11], components of the TGF-β pathway instrumental in fine-tuning proliferation for efficient erythropoiesis [12]. We provide multiple lines of experimental evidence to show that this microRNA modulates TGF-β signaling by directly inhibiting SMAD2 and SMAD4 production, thus promoting proliferation during human *in vitro* erythroid differentiation. Our study is the first to comprehensively profile the erythrocyte transcriptome, and reflects the utility of high-throughput sequencing to identify critical modulators of human development.

## Results

### Mature erythrocytes contain a diverse repertoire of long RNAs

**Fig. 1:**
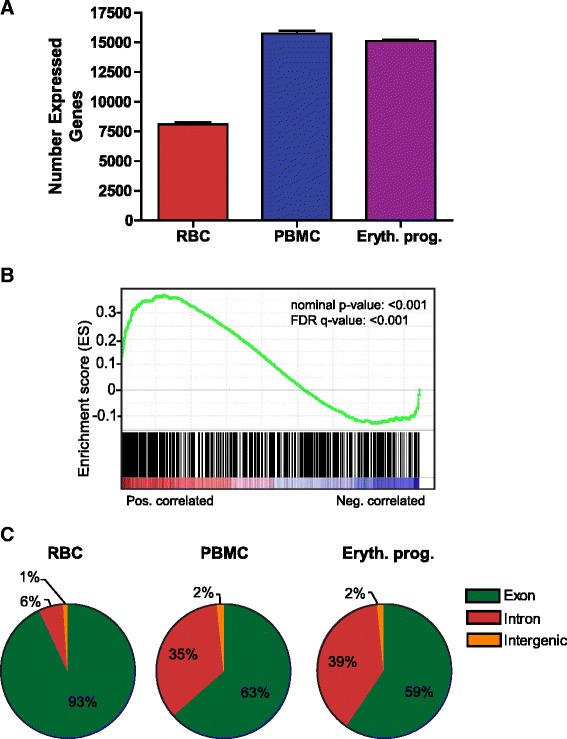
Population characteristics of erythrocyte long RNAs. **a** Distribution of total number of expressed transcripts across indicated cell types. A transcript was considered expressed if the RPKM value was ≥0.5. **b** GSEA analysis of the top 500 expressed erythrocyte transcripts in the ranked expressed transcripts of the day 8 erythroid progenitor (D8) vs. PBMC samples. **c** Distribution of the genomic location for RNA-Seq reads. Locations represent average number of reads for the three RBC, three PBMC, and two erythroid progenitor independent samples. Intronic reads are included if <10 kb upstream of a transcription start site or <10 kb downstream of a transcription end site

To extensively profile the complete transcriptome of mature erythrocytes, we obtained highly purified erythrocytes from healthy donors. As previously described [13], blood samples were leukocyte-depleted, separated using a density gradient, and CD71- mature erythrocytes were magnetically-selected. The purity of the sample was first verified by flow cytometric analysis of CD71 expression (Additional file 1: Figure S1A) and further validated by the lack of leukocyte transcripts in the sequencing data (described later). We isolated total RNA, including small-sized RNA, and constructed sequencing libraries for both short (18–24 nt) and long (>200 nt) RNAs from erythrocyte RNA samples. RNA from five individuals was used for erythrocyte short RNA-seq, and RNA from three individuals was used for erythrocyte long RNA-seq. Additionally, we isolated total RNA from peripheral blood mononuclear cells (PBMCs) of three individuals, and RNA from *in vitro* differentiating CD34+ erythroid progenitors (Day 8 of differentiation) of two individuals. RNA from these nucleated erythroid and peripheral blood mononuclear cells was isolated and used to prepare strand-specific long RNA-seq libraries (detailed in Methods) to compare with the transcriptome of erythrocytes. For long RNA-seq, hemoglobin and ribosomal RNAs were first depleted from the sample, then barcoded sequencing libraries were generated using random primers. The sequencing libraries were pooled and 50 bp paired-end sequencing was performed using the Illumina HiSeq 2000 system. While we did not expect abundant long RNAs in mature erythrocytes, sequencing unexpectedly identified a large, diverse repertoire of long RNAs in erythrocytes. The 25 most abundant erythrocyte transcripts (Table 1) and entire catalog (Additional file 2: Table S1) of expressed long RNAs are described. To determine both shared and unique aspects of the erythrocyte transcriptome, we compared the erythrocyte transcriptome with that of the PBMC and CD34+ erythroid progenitor transcriptomes. Libraries from these nucleated cells were prepared and run in parallel to that of the erythrocyte long RNA sequencing samples. Using the same analytic methodology and threshold (RPKM of ≥0.5), we found that mature erythrocytes had far fewer expressed genes (~8092 genes) than other nucleated blood cells such as PBMCs (~15743 genes) and erythroid progenitors (~15113 genes) (Fig. 1a). However, mature erythrocytes still have thousands of transcripts that may provide unique insights into erythroid biology.

**Table 1:** Top 25 expressed long RNAs in erythrocytes

Rank	Gene symbol	Description	Average relative expression
1	RNY1	RNA, Ro-associated Y1	847620
2	RNY4	RNA, Ro-associated Y4	215019
3	RPPH1	ribonuclease P RNA component H1	96625
4	RN7SK	RNA, 7SK small nuclear	41008
5	UBA52	Ubiquitin A-52 residue ribosomal protein fusion product 1	15576
6	UBB	Ubiquitin B	15389
7	BNIP3L	BCL2/adenovirus E1B 19 kDa interacting protein 3-like	14362
8	SLC25A37	Solute carrier family 25 (mitochondrial iron transporter), member 37	12246
9	FTL	ferritin, light polypeptide	10475
10	SNCA	synuclein, alpha (non A4 component of amyloid precursor)	9959
11	TMEM56	transmembrane protein 56	8918
12	RPS12	ribosomal protein S12	7359
13	EPB41	erythrocyte membrane protein band 4.1 (elliptocytosis 1, RH-linked)	6337
14	GYPC	glycophorin C (Gerbich blood group)	5744
15	RN7SL4P	RNA, 7SL, cytoplasmic 4, pseudogene	4953
16	AC079949.1	no description available	4543
17	PICALM	Phosphatidylinositol binding clathrin assembly protein	4443
18	OAZ1	Ornithine decarboxylase antizyme 1	4330
19	ALAS2	5,-aminolevulinate synthase 2	4289
20	MBOAT2	Membrane bound O-acyltransferase domain containing 2	4250
21	RNY5	RNA, Ro-associated Y5	4027
22	FBXO7	F-box protein 7	4008
23	DNAJC6	DnaJ (Hsp40) homolog, subfamily C, member 6	3660
24	ADIPOR1	Adiponectin receptor 1	3574
25	SERF2	small EDRK-rich factor 2	3398

To determine whether the long RNA erythrocyte transcriptome reflects that of erythroid progenitors versus PBMCs, we used GSEA (Gene Set Enrichment Analysis) to determine the relative enrichment of the top 500 erythrocyte RNA transcripts in the day 8 erythroid progenitor (D8) vs. PBMC samples. We observed a highly significant enrichment of the top 500 erythrocyte transcripts in the erythroid progenitor transcriptome (Fig. 1b). Together, our data shows selective retention of many long RNAs previously transcribed in nucleated erythrocyte progenitors, consistent with the possibility the erythrocyte RNAs were derived from erythroid precursors.

Recent studies have suggested that intron retention and nonsense mediated decay may contribute to degradation of most transcripts during terminal differentiation of granulocytes [14] and erythrocytes [15]. Therefore, we analyzed the relative distribution of gene mapping regions for erythrocyte long RNAs. On average, 93 % of human erythrocyte long RNAs map to annotated exons of coding and noncoding RNAs, far higher than that of PBMCs (63 %) and erythroid progenitors (59 %) (Fig. 1c). Therefore, compared with nucleated cells, relatively few erythrocyte transcripts map to introns and intergenic regions. This difference may reflect that nucleated cells, when compared with anucleate erythrocytes, retain more unprocessed RNAs in the nucleus. We also observed slightly less coverage at the 3’ of erythrocyte transcripts, compared to that of PBMC and erythroid progenitor transcripts (Additional file 1: Figure S1B). Additionally, when the top 500 highest expressed erythrocyte genes were examined for GO (gene ontology) enrichment, we found significant enrichment in pathways of cellular, protein and macromolecule biosynthesis and metabolisms, defense response, and protein ubiquitination (Additional file 3: Table S2). These categories represent both known and less appreciated functions critical for erythrocyte activities, such as protein and macromolecule synthesis for hemoglobin. Ubiquitin was originally identified in erythrocytes [16], and ubiquitination is essential for enucleation during erythrocyte development [17]. In addition, the enriched categories include GO of G-protein coupled receptor (GPRC) protein signaling pathways and defense response. Several studies have highlighted the role of GPCRs in erythropoiesis [18, 19]. The enrichment of GO in defense response is unexpected and deserves investigation in future studies.

We also did not identify enrichment for inflammatory-related gene signatures typically found in leukocytes samples. None of the transcripts that encode leukocyte marker such as CD20 (MS4A1) and different CD3 mRNAs, were expressed in any erythrocyte sample. However, these transcripts were identified in the PBMC transcriptomes. Together, this sequencing data validates the successful depletion of leukocytes in the purified erythrocyte samples.

Next, we examined the most abundant transcripts to obtain insights into erythroid biology. Several of the most highly expressed RNAs are Y RNAs or different components of ribonucleoprotein complexes that represent persistent expression after terminal differentiation. Many of the highest expressed genes in the erythrocyte transcriptome also encode proteins highly relevant for erythroid cell differentiation. For example, BNIP3L mediates mitochondrial clearance during reticulocyte terminal differentiation [20]. Abundant SLC25A37 encodes mitoferrin-1, an essential iron importer for the synthesis of mitochondrial heme and iron-sulfur clusters in erythroblasts [21], and FLT encodes the ferritin light chain, a major component of intracellular iron storage [22]. Another top expressed gene, EPB41, constitutes the red cell membrane cytoskeletal network, which plays a critical role in erythrocyte shape and deformability [23].

In addition, several highly expressed erythrocyte transcripts encode proteins with known functions previously not associated with erythrocytes. For example, ADIPOR1 encodes the cellular receptor for adiponectin, a hormone secreted by adipocytes that regulates fatty acid catabolism and glucose levels. Interestingly, Japanese individuals with anemia present significantly higher serum levels of adiponectin that that of unaffected individuals, yet the function of such an association remains unknown [24]. Highly expressed TMEM56 (Transmembrane protein 56) and OAZ1 (Ornithine Decarboxylase Antizyme 1) may also have undiscovered roles in erythroid biology. The discovery of these unexpected genes prompts future functional investigation and may provide unexpected insights into erythroid biology.

### Mature erythrocytes contain a diverse repertoire of known and putative microRNAs

**Fig. 2:**
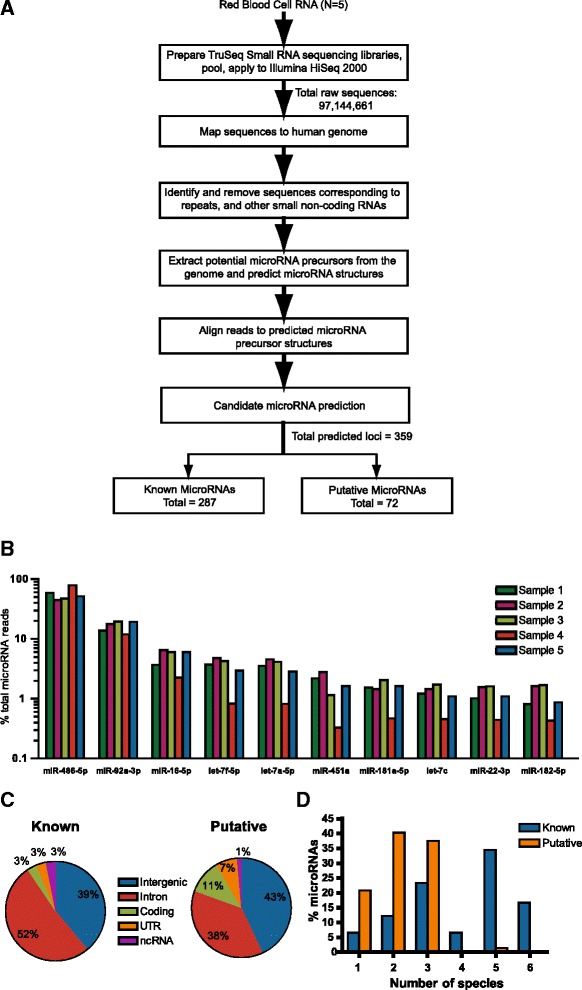
MicroRNA identification pipeline and microRNA population characteristics. **a** Deep sequencing of short RNAs from mature RBCs was performed using the Illumina HiSeq technology. Raw sequences were mapped to the genome, filtered through miRDeep, and categorized. Identified microRNA loci were cross-referenced with miRBase (v. 21) and UCSC Genome Browser (hg 38) to distinguish between candidate novel and known microRNAs. **b** Relative percentages (log base 10) of read numbers for top 10 microRNAs among five different samples. Percentages are averages based on total number of known, mature microRNA reads for each sample. **c** Relative percentage representation of genomic location for both known and putative microRNAs. Listed genomic locations for mature microRNA sequences are relative to RefSeq annotated transcripts in UCSC genome browser (hg38). For mature microRNA sequences spanning both introns and coding region, or UTR and coding regions of alternative transcripts, sequence is listed in coding region. **d** Relative percentage representation of sequence conservation for both known and putative microRNAs. Conservation of mature microRNA sequence indicates exact sequence alignment in listed number of species (from human, chimp, rhesus monkey, dog, mouse, and zebrafish), allowing for up to one base mismatch outside of the microRNA seed sequence (nucleotides 2–8), and no mismatches within the seed sequence

In parallel with long RNAs, we also sequenced short (18–24 nt) RNA species from five erythrocyte samples. The short RNA libraries were constructed, pooled, and then applied to the Illumina HiSeq 2000 to generate a total of 97,144,661 reads. To discover small-sized RNAs with microRNA-like characteristics, we employed the miRDeep2 pipeline [25] (Fig. 2a), a widely used [26, 27] probabilistic model algorithm based on canonical microRNA precursor processing, and is therefore not limited to previously annotated or highly conserved microRNAs.

We employed a cutoff for microRNAs based on a miRDeep score of ≥1, and ≥ 20 reads in at least one sample, like that previously used [28]. In all, we identified 359 microRNAs. For the top 10 identified microRNAs, similar percentages of reads were found in all five erythrocyte samples (Fig. 2b), indicating reproducible expression of the most highly expressed microRNAs. All expressed known microRNAs (Additional file 4: Table S3) and all putative microRNAs (Additional file 5: Table S4) are shown.

Several of the most abundant microRNAs in our dataset were previously shown to be enriched in human erythrocytes, thus validating our findings. For example, the most abundant microRNA, miR-486-5p, accelerates erythroid differentiation [29], and its overexpression was associated with an erythroid-like subtype of megakaryocytic leukemia [30]. MiR-451a and miR-144-3p are both GATA-1-responsive microRNAs upregulated during erythroid differentiation and play important roles in the anti-stress capacity of erythrocytes [7, 8, 31–34]. Additionally, miR-16, miR-92a, and the let-7 family of microRNAs are all among the most abundant erythrocyte microRNAs from previous studies [4, 35, 36]. Importantly, high plasma levels of miR-486-5p, miR-92a, miR-16 and miR-451a are associated with increased red cell hemolysis in human plasma samples, consistent with their high abundance in erythrocytes [37, 38]. Hence, our sequencing data identified a large number of erythrocyte microRNAs. While most of these microRNAs were previously associated with erythrocytes, several abundant microRNAs, such as miR-182-5p, have not been previously associated with erythroid cells.

The putative microRNAs identified by miRDeep were further manually annotated and curated using both the UCSC genome browser (hg38) and miRBase (v.21). These analyses lead to 72 putative unannotated microRNAs, and selected microRNA sequences that met criteria for putative microRNAs are shown (Additional file 1: Figure S2). We then determined the relative genomic location and sequence conservation for both known and putative erythrocyte microRNAs. The majority of both known (91 %) and putative (81 %) microRNAs mapped to intergenic or intronic regions, with few microRNAs mapped to coding, untranslated, or long ncRNA regions (Fig. 2c). These results indicate that overall, newly identified putative erythrocyte microRNAs reside in similar genomic regions to that of known microRNAs. We also examined the evolutionary conservation of both known and putative microRNAs across six species (human, chimp, rhesus monkey, dog, mouse, and zebrafish) by the number of species in which each full, mature microRNA sequence was found (Fig. 2d). Known microRNAs were identified in more species (4–6 species) than putative microRNAs (1–3 species), and were therefore more conserved. Additionally, most putative microRNA sequences (99 %) were only identified in primates, while far fewer known microRNAs were only identified primates (42 %). This disparity highlights the utility of miRDeep to identify putative microRNAs that are mostly primate-specific.

### Joint analysis of erythrocyte long and short RNA transcriptomes reveals novel elements within the miR-144/451 locus

Next, we performed a joint analysis of the long and short RNA erythrocyte transcriptomes. To determine whether erythrocyte microRNAs may post-transcriptionally regulate erythrocyte mRNAs, we assessed whether these microRNAs may contribute to selective enrichment or depletion of erythrocyte mRNAs. GSEA was used to evaluate the potential enrichment and depletion of the predicated target mRNAs for the top six expressed erythrocyte microRNAs: miR-486-5p, miR-92-3p, miR-16-5p, let-7a/f, and miR-451 in erythrocyte mRNAs, compared to that of PBMC mRNAs. We observed that the target mRNAs of these top expressed miRNAs were not significantly enriched or depleted in the erythrocyte mRNA dataset (Additional file 1: Figure S3).

**Fig. 3:**
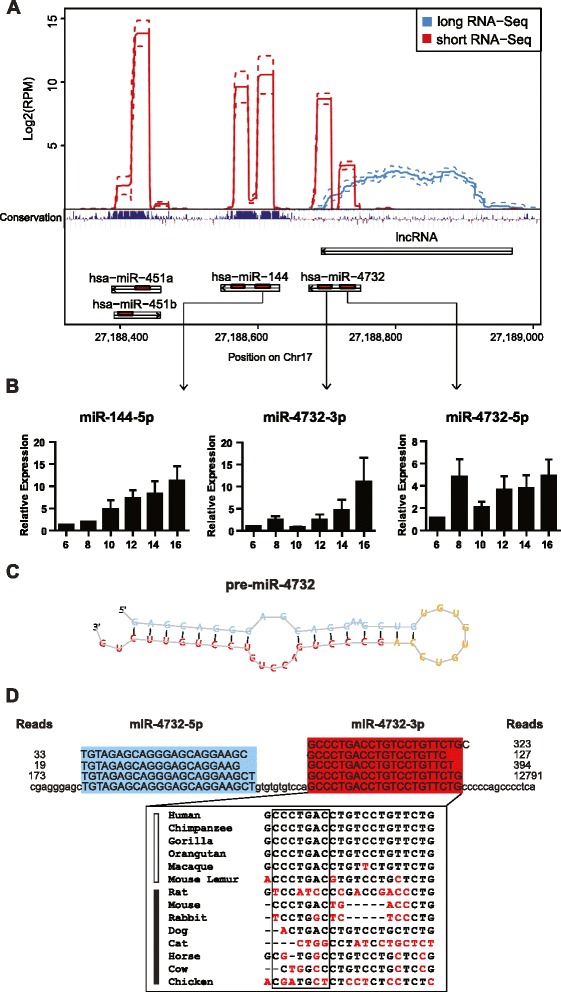
Genomic locations and expression dynamics of noncoding RNAs in the miR-144/451 locus. **a** Location, sequence read length, relative read number, and vertebrate conservation of noncoding RNAs within the chromosome 17 miR-144/451 locus according to UCSC genome browser (hg38). Solid lines indicate average read numbers, dotted lines represent highest and lowest standard deviations of read numbers across the samples. The strand orientation of each noncoding RNA is indicated. **b** Expression of miR-144-5p, miR-4732-3p, and miR-4732-5p, during CD34+ erythroid differentiation using progenitors from three different individuals. Differentiation is listed from day 6 to day 16. U6 snRNA was used as a loading control, with fold difference set relative to day 6. **c** Predicted folding structure for the miR-4732 pre-microRNA according to miRDeep, and frequency of reads for mature microRNAs from one representative sample. The most prevalent mature read sequences for miR-4732-3p (red) and miR-4732-5p (blue) are shown. **d** Sequence conservation of miR-4732-3p in listed species. Primates are listed adjacent to white box, non-primates listed adjacent to black box. Seed sequence is boxed, and base mismatches with human miR-4732-3p are highlighted in red

Next, we identified long and short RNAs that map to adjacent regions. Several long RNA transcripts that spanned different annotated pre-microRNA loci were identified (Additional file 6: Table S5). Together, we observed three instances of co-expression for both erythrocyte microRNAs and long RNAs spanning pre-microRNA loci. There are two distinct patterns of co-expression. In the first pattern, we observed co-expression of both microRNAs and long RNA transcripts spanning entire pre-microRNAs, including 5’ and 3’ flanking regions in two loci (miR-6087 and miR-3687). In the second pattern, we observed co-expression a long RNA spanning only the 5’ portion of the miR-4732 pre-microRNA, proximal to the miR-144/451 region; this lncRNA was confirmed via RT-PCR (Additional file 1: Figure S4). We focused on the co-expressed microRNAs and the long RNA within the miR-144/451 locus for several reasons (Fig. 3a). This locus has been widely implicated in erythropoiesis, and all RNAs in this locus contain a much higher coverage (>50 reads) than that of RNAs in other co-expression loci. Interestingly, when the sequencing reads were mapped to the miR-144/451 locus, we found several RNA reads mapped to distinct elements 5’ of pre-miR-144. These RNAs include a ~250 nt lncRNA spanning the 5’ hairpin region of pre-miR-4732, as well as miR-4732-5p and -3p (Fig. 3a).

Among the newly identified erythrocyte RNAs, we investigated the functional role of miR-4732-3p for several reasons. First, both miR-4732-5p and -3p are previously uncharacterized. Second, miR-4732-3p is much more highly expressed than miR-4732-5p. Additionally, proximal microRNAs miR-451a and miR-144-3p are significantly induced during erythroid differentiation [8]. We used real-time PCR to determine the expression dynamics of miR-4732-3p and other erythrocyte microRNAs at different time points during *in vitro* differentiation of CD34+ erythroid progenitors (Fig. 3b). Similar to miR-451a and miR-144-3p [8], both miR-144-5p and miR-4732-3p were also upregulated during erythropoiesis (Fig. 3b). However, miR-4732-5p levels were less dramatically upregulated during erythropoiesis. Additionally, miR-486-5p was upregulated and miR-221 was downregulated during differentiation, as previously described [29, 39] (Additional file 1: Figure S5), indicating successful differentiation of our *in vitro* erythroid culture. Upregulation of miR-4732-3p, as well as its physical proximity to the miR-144/451 locus suggests its similar regulation and functional role during erythroid development.

MirDeep maps miR-4732 to a predicted pre-microRNA canonical stem-loop folding structure with distinct 5p- and 3p- mature sequence reads (Fig. 3c), indicative of a *bona fide* microRNA. Interestingly, miR-4732-3p is primate-specific, with full seed sequence identical only among primates. Within non-primate species, it is poorly conserved, with both seed and non-seed mismatches (Fig. 3d). In contrast, miR-451a, miR-144-3p, and miR-144-5p are all highly conserved among primate and non-primate species (Additional file 1: Figure S6). Together, the significant miRDeep score (1.9), predicted pre-microRNA structure, and proximity to the miR-144/451 locus underscores the authenticity and potential functional relevance of miR-4732-3p.

### MiR-4732-3p directly regulates SMAD2 and SMAD4

Using the microRNA target prediction tool Targetscan [11], 556 genes were predicted to be regulated by miR-4732-3p (Additional file 7: Table S6). We performed a GATHER analysis [40] to identify the related predicted target pathways among the target genes. We identified significant enrichment in TGF-β signaling, with literature networks and protein-binding networks for SMAD3 [40]. SMAD2 and SMAD4 are both predicted to be regulated by miR-4732-3p, with conserved seed sequence binding sites in most species. TGF-β family cytokines bind to associated membrane receptors, promoting receptor-mediated phosphorylation of SMAD2 and SMAD3 to associate with SMAD4 [12]. This activated SMAD2/3 complex binds to competing effectors SMAD4 or TIFγ to fine-tune a balance between erythroid differentiation and proliferation [41]. TGF-β activates the expression of several SMAD4 target genes, including *pai-1*, *p21*, *bim*, and *bax* [42, 43], shown to reduce cell viability and proliferation. For example, PAI-1, Bim, and Bax act as activators of apoptosis and trigger cell death [44, 45]. In addition, p21 is a potent cell cycle inhibitor that binds to and inhibits the activity of cyclin-CDK2, −CDK1, and -CDK4/6 complexes [46]. The induction of these genes by TGF-β is consistent with ability of TGF-β to inhibit proliferation and increase cell death during erythropoiesis [47]. Inhibition of SMAD2 or SMAD4 results in increased erythroid cell proliferation [43, 48–51], but their effects on differentiation are inconsistent. However, much remains unknown about the upstream signals that coordinate the regulation of these factors.

**Fig. 4:**
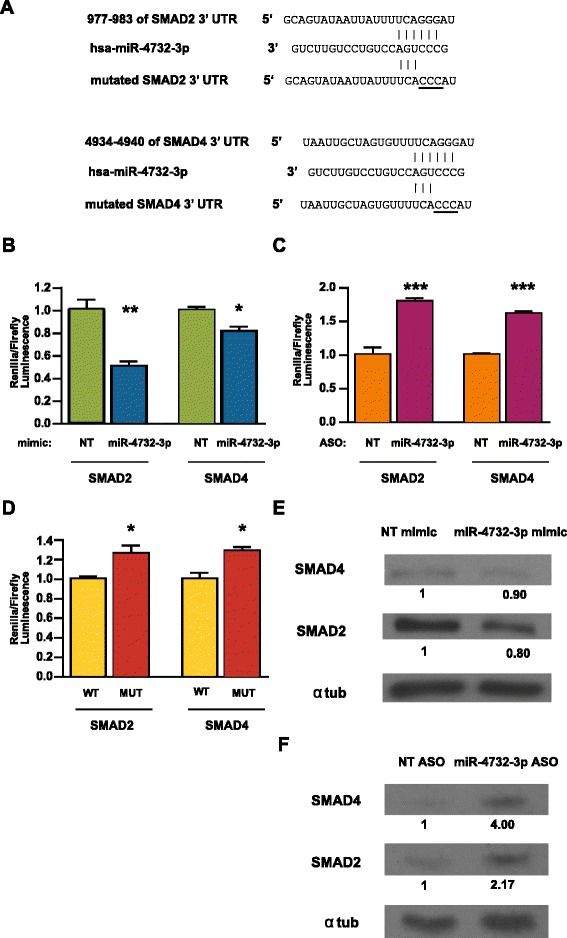
MiR-4732-3p directly regulates SMAD2 and SMAD4. **a** Alignment of miR-4732-3p and predicted target sites in the 3’ UTR of SMAD2 and SMAD4, with mutated binding sites underlined. **b** Relative changes of reporter activities in K562s co-transfected with luciferase constructs and indicated microRNA mimics. A portion the SMAD2 and the SMAD4 3’UTRs were cloned downstream of Renilla in separate psiCheck-2 vectors (*n* = 4) (*p < 0.05, **p < 0.01). **c** Relative changes of reporter activities in K562s co-transfected with luciferase constructs and microRNA-blocking antisense oligonucleotides (ASOs) (*n* = 4) (***p < 0.001). **d** Relative changes of reporter activities in K562s transfected with wild-type (WT) or mutated (MUT) SMAD2 or SMAD4 3’UTR constructs (*n* = 4) (*p < 0.05). **e** Western blots of SMAD2 and SMAD4 protein expression 24 h after transfection of K562s with indicated microRNA mimics or **f** microRNA ASOs. α-tubulin was used as a loading control for normalization. Protein desitometric values are listed. The fold change of expression for each treatment group was first normalized to alpha tubulin input for that treatment, then ratio set relative to the control treatment

Based on these predictions and biological relevance, we investigated the potential of miR-4732-3p to regulate SMAD2 and SMAD4 (Fig. 4a). To test whether both of these predicted targets contain *bona fide* repressive elements, portions of 3’UTR of SMAD2 and SMAD4, including the predicted target sites, were cloned into a dual luciferase reporter. As shown, transfection with a miR-4732-3p mimic significantly repressed normalized Renilla 3’ UTR reporter activities of both SMAD2 and SMAD4 in K562s, indicating the ability of miR-4732-3p to regulate SMAD2/4 (Fig. 4b). To determine whether endogenous miR-4732-3p repression would affect reporter expression, we inhibited miR-4732-3p with an antisense oligonucleotide (ASO) and observed increased relative Renilla reporter activities for both SMAD2 and SMAD4 (Figs. 4c). This regulation occurs through the predicted target miR-4732-3p binding sites, as mutation of the respective target sequences in the 3’ UTR of SMAD2 and 4 abrogated the endogenous microRNA-mediated luciferase repression (Fig. 4d).

To understand the *in vivo* relevance of the regulatory relationship between miR-4732-3p and SMAD2/SMAD4, we determined whether miR-4732-3p inhibits the protein levels of SMAD2 and SMAD4 (Fig. 4e-f). Transfection of miR-4732-3p mimics in K562s reduced the protein levels of both SMAD2 and SMAD4 (Fig. 4e), whereas inhibition of miR-4732-3p increased the levels of SMAD2 and SMAD4 protein (Fig. 4f). Thus, miR-4732-3p inhibits the protein levels of both SMAD2 and SMAD4 through a canonical microRNA-mediated regulation of 3’ UTRs.

### miR-4732-3p regulates the TGF-β signaling cascade

**Fig. 5:**
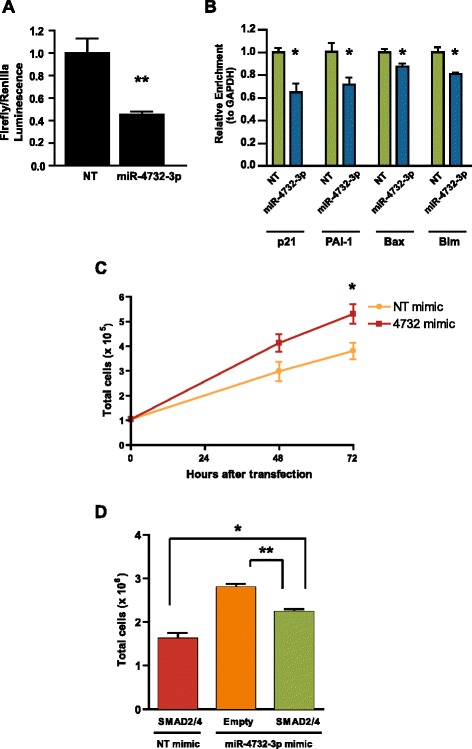
MiR-4732-3p regulates TGF-β signaling and promotes erythroid cell proliferation. **a** Relative changes of TGF-β reporter activities in K562 cells co-transfected with luciferase constructs and microRNA mimics (*n* = 4) (**p < 0.01). Cells were transfected with microRNA mimic, SMAD4-responsive Firefly construct, and a separate Renilla construct as a transfection control. **b** Relative mRNA levels of p21, PAI-1, Bax, and Bim 24 h after transfection of K562s with microRNA mimics. Levels were determined using qPCR and normalized by GAPDH (*n* = 3) (*p < 0.05). **c** Total cell number of primary CD34 erythroid progenitors 48 and 72 h after equal numbers of cells were transfected with indicated microRNA mimics on day 8 of differentiation (*n* = 3) (*p < 0.05). **d** Total cell number of primary CD34 erythroid progenitors 24 h after equal numbers of cells were transfected with indicated combinations of microRNA mimics and overexpression constructs on day 8 of differentiation (*n* = 3) (*p < 0.05, **p < 0.01)

Next, we assessed whether miR-4732-3p-mediated downregulation of SMAD2 and SMAD4 would affect TGF-β signaling. TGF-β pathway activities were measured with a Firefly luciferase TGF-β reporter construct driven by the SMAD4-dependent CAGA element promoter (CAGA-luc) [52]. Overexpression of miR-4732-3p in K562s resulted in a significant reduction in relative reporter luciferase values (Fig. 5a), demonstrating that miR-4732-3p suppresses SMAD4-mediated transcription activities. Additionally, we determined whether miR-4732-3p overexpression may affect the level of SMAD4-dependent target genes, such as *pai-1*, *p21*, *bim*, and *bax* [42, 43]. Overexpression of miR-4732-3p significantly reduced the expression of all these SMAD4-regulated genes (Fig. 5b).

Given the ability to suppress TGF-β signaling activity, we hypothesized that miR-4732-3p may promote proliferation by suppressing SMAD2 and SMAD4. Consistent with this hypothesis, overexpression of miR-4732-3p in differentiating CD34+ erythroid progenitors resulted in a significant increase in total cell number (Fig. 5c). Furthermore, the miR-4732-3p-mediated increase in cell number was abolished by overexpression of SMAD2 and SMAD4 with cDNA constructs lacking miR-4732-3p-responsive 3’UTRs (Fig. 5d). These results indicated that miR-4732-3p increased erythroid cell number by repressing the levels of SMAD2/4. Together, these data are consistent with the possibility that miR-4732-3p modulates TGF-β signaling to promote erythroid cell survival and production during erythropoiesis.

## Discussion

The discovery of abundant and diverse erythrocyte RNAs opens the possibility of using these readily accessible genetic materials as biomarkers to monitor various human diseases and treatments that involve erythroid cells. Sequencing allows for characterization and quantification of not only annotated RNAs, but also novel mRNA isoforms and noncoding RNAs. To the best of our knowledge, this is the first study that has employed high-throughput sequencing to extensively profile both the long and short RNA transcriptomes of human erythrocytes.

Here we focus on the primate-specific miR-4732-3p. While this microRNA has been annotated in miRBase (v.21), it has few reads (<10 per sample) in other datasets and is uncharacterized. We found that miR-4732-3p is an abundant erythrocyte microRNA within the erythroid-enriched miR-144/451 locus. The identification of multiple elements within the miR-144/451 cluster is not surprising, as miR-144 and miR-451 are both highly expressed and upregulated by GATA1 during erythrocyte development [8]. Considering the proximity of these newly identified noncoding RNAs to this locus, these RNAs are also likely upregulated by GATA1. In addition, the lncRNA overlapping miR-4732 may exist as a truncated form of a longer RNA precursor which undergoes further processing for production of the adjacent microRNAs within this locus. Conversely, this lncRNA may simply be overlapping within this region and not subject to coordinated processing and regulation of adjacent noncoding RNAs. We suggest further investigation into the potential RNA processing mechanisms within this locus and functions of these noncoding RNAs in erythroid cells.

The human erythrocyte is one of the most commonly studied cell types, often used as a model system to understand the general principles of molecular genetics, biochemistry, membrane biology, and cell physiology. As new RNA synthesis ends with nuclear extrusion, the expression level of any RNA species is determined mainly by its turnover and decay [53]. The selective persistence of particular RNAs in mature erythrocytes likely reflects associated decay kinetics. Given the relative stability of microRNAs, plasma microRNAs have been proposed as valuable biomarkers. Several studies have suggested that select plasma microRNAs are highly associated with hemolysis. Many erythrocyte microRNAs, including miR-92a and miR-486-5p, have been proposed as candidate plasma biomarkers for human diseases, yet red cell lysis may contribute to this association, a concern that has been raised previously [38].

As many microRNAs are highly conserved between species [54], few primate-specific microRNAs have been characterized [55, 56]. Primate-specific microRNAs are generally associated with brain function, immune response, or cancer progression. Our study is the first to identify the role of a primate-specific microRNA in hematopoiesis. Mir-4732-3p provides an additional layer of regulation for primate erythropoiesis. Although mouse and other non-primate models have provided valuable insight into human physiology and disease, considerable divergence still exists between human and mouse erythropoiesis [57]. Notably, several groups have observed mild anemic phenotypes with deletion of the abbreviated miR-144/451 locus in mice, yet these studies did not take into account potential regulatory factors flanking this site [31, 58]. Our study further illustrates the discrepancy between the regulatory mechanisms of human and mouse erythropoiesis and supports investigation specifically within human models to consider mechanisms that are not evolutionarily conserved.

In this study, we demonstrated that miR-4732-3p inhibits TGF-β signaling to promote erythroid cell expansion during differentiation. As inhibition of SMAD2 and SMAD4 via RNAi-mediated mechanisms reflects phenotypes previously documented [12, 43], this microRNA modulates SMAD2 and SMAD4 activities to achieve similar phenotypes. Dysregulation of the TGF-β pathway has been implicated in several human erythroid diseases. For example, overactivation of TGF-β signaling has been extensively reported in β-thalassemia and MDS (myelodysplastic syndrome), both characterized by ineffective erythropoiesis and anemia [50, 51, 59]. Though other mRNA targets and phenotypes associated with miR-4732-3p have yet to be elucidated, miR-4732-3p represents a unique, endogenous TGF-β signaling inhibitor that promotes erythroid expansion, and has the potential to be manipulated as a biomarker or novel therapeutic for various anemia disorders.

## Conclusions

Here we describe the first comprehensive, as well as joint analysis of the human erythrocyte transcriptome. We have made several important observations. First, in addition to microRNAs, mature erythrocytes also contain long transcripts. Although smaller in number, erythrocyte long transcripts are highly enriched in annotated exons and largely encode proteins critical to erythrocyte differentiation and functions. Therefore, quantitative analysis of the erythrocyte transcriptome may reflect erythrocyte development history and pathological adaptations. Second, miRDeep identified 287 known and 72 putative novel microRNAs that include many microRNAs that are either uncharacterized, or not previously associated with erythrocytes. Third, the joint analysis of long and short transcripts identified a long noncoding RNA and previously uncharacterized microRNAs in the miR-144/451 locus. Finally, functional studies demonstrate the primate-specific microRNA miR-4732-3p regulates the TGF-β pathway during human erythropoiesis. This study presents a novel post-transcriptional modulator of erythroid development, and features a new transcriptomic data set that provides new insights into erythroid biology.

## Methods

### Ethics, consent, and permissions

Blood samples were obtained from healthy adults via venipuncture into vacutainer tubes (BD) using a protocol (Pro00050295) approved by the Institutional Review Board at Duke University Medical Center. Written, informed consent was obtained from each donor.

### Consent to publish

We have obtained consent to publish from the blood donors to report individual donor data.

### Cell sorting, purification, and RNA isolation

Blood was kept on ice after a single draw and was processed within 30 min of sample acquisition. For isolation of erythrocytes from whole blood, blood samples were processed as previously described [13]. Briefly, a portion of whole blood was washed three times with PBS, centrifuged, and plasma and buffy coat removed. Leukocytes were then depleted from the sample using a Purecell® Leukocyte Reduction Filtration System. Reticulocytes (immature red blood cells) were removed in the CD71 positive fraction using CD71 microbeads with the autoMACS® Separator (Miltenyi Biotec). For confirmation of RBC purity after magnetic bead sorting, we used a CD71-PE antibody (Miltenyi Biotec). Flow cytometry was run on the BD FACSCantoII system using the FACSDiva software. All additional flow cytometric analyses were performed using the FlowJo (v.7.6.4) software. For isolation of PBMCs, a buffy coat was separated from whole blood using a Ficoll-Paque (GE Healthcare) centrifugation gradient, and remaining erythrocytes were lysed using a red cell lysis buffer (Qiagen). Total RNA was isolated using the mirVANA miRNA isolation kit (Ambion).

### RNA library preparation and sequence analysis

To prepare long RNA‐Seq libraries, 1 μg of purified total input RNA was used with the TruSeq Stranded Total RNA Sample Prep Kit with Ribo‐Zero Globin (Illumina). A total of 100 ng of erythrocyte RNA was used to prepare short RNA-seq libraries with the TruSeq Small RNA Sample Prep Kit (Illumina). Total RNA-seq and small RNA libraries were separately pooled and analyzed using the Illumina HiSeq 2000 sequencing system. All reads for total RNA-seq and small RNA-seq were sorted based on quality, and raw sequences with removed 3’ linker sequences were obtained in FASTQ format. All primary sequencing data are publicly available through the Gene Expression Omnibus (GEO dataset GSE63703). For long RNA-seq, the RNA-seq data was processed using the TrimGalore toolkit to trim low quality bases and Illumina sequencing adapters from the 3’ end of the reads. Only pairs where both reads were 20 nt or longer were kept for further analysis. Reads were mapped to the GRCh37r73 version of the human genome and transcriptome [60] using the STAR RNA-seq alignment tool [61]. Reads were kept for subsequent analysis if they mapped to a single genomic location. RNA-Seq quality control, genomic position summary statistics, and gene body coverage statistics were generated with the RSeQC toolkit [62]. Gene expression in reads per million per kilobase (RPKM) value were calculated using the cufflinks algorithm [63]. Genes that had an RPKM value ≥0.5 in at least one sample were kept for subsequent analysis. Short RNA-seq bioinformatic analyses were performed using miRDeep2 and in house Perl scripts, with methods similar to that previously described [28]. Gene set enrichment analysis (GSEA) [64] was computed using the top 500 genes expressed in RBCs as the ‘gene set’ , and the log2-fold-change of the D8/PBMC samples as the input list to test for the enrichment of top RBC expressing transcripts against those more specific to D8 samples or the PBMC samples.

### Cell culture

Mobilized Peripheral Blood CD34+ Cells (AllCells) were grown in StemLine II media containing Glutamine (Sigma) with 20 % BIT (StemCell Technologies) and 1 % Penicillin/Streptomycin (Gibco). On Days 0–7, cells were supplemented with growth factors IL-3 at 20 ng/mL (Invitrogen), SCF at 50 ng/mL (Invitrogen), and Erythropoietin (Epo) at 3U/mL (Calbiochem). On days 8–12 cells were only supplemented with IL-3 and Epo, and on days 13–16 cells were only supplemented with Epo. K562 cells (ATCC) were maintained in RPMI containing glutamine (Gibco), with 10 % Fetal Bovine Serum (Hyclone) and 1 % Penicillin/Streptomycin (Gibco). All cell counts were performed using a standard hemocytometer.

### Transfection and luciferase assays

K562 and CD34 erythroid progenitor cells were transfected with indicated plasmids and/or oligos using Lipofectamine LTX (Invitrogen), HiPerfect (Qiagen), or Attractene (Qiagen) reagents. For luciferase values, all assays were performed in biological quadruplicate, and Firefly and Renilla luciferase activities were measured using the Dual-Glo Luciferase assay (Promega) and luminometer (Tecan Infinate F200).

### Western blot analysis

Cells were lysed with RIPA buffer supplemented with protease inhibitor cocktail (Sigma). Denaturing sample buffer was added to protein samples, and samples were boiled and resolved on Tris–HCl polyacrylamide gels. Resolved proteins were then transferred onto polyvinylidene fluoride (PVDF) membranes (GE Healthcare) and probed with the following primary antibodies: anti-rabbit Smad2 and anti-rabbit Smad4 (Cell Signaling Technology, Inc.), and anti-mouse α-Tubulin (Sigma). The following secondary antibodies were used: goat anti-rabbit IgG-HRP (Santa Cruz) and anti-mouse IgG-HRP (R&D Systems). The western blot was visualized by enhanced chemiluminescence (Western Lightning-ECL Plus, PerkinElmer) and exposed to film. Densitometric measurements were performed with ImageJ software (http://rsb.info.nih.gov/ij/).

### Quantitative PCR

To quantify relative microRNA expression, 200 ng of total RNA was reverse-transcribed using the Taqman microRNA reverse transcription kit (ABI). The resulting cDNA was used to assess RNA expression by qPCR with TaqMan microRNA real-time assays and Taqman universal master mix (ABI). Results were calculated using the comparative CT method. We compared relative levels of microRNAs with probes specific for the indicated mature microRNA and normalized by U6 snRNA expression. To quantify relative mRNA expression, RNA was reverse-transcribed with SuperScript II following the manufacturer’s protocol (Invitrogen). The resulting cDNA was used to assess RNA expression by qPCR with Power SYBRGreen PCR mix (ABI) and primers specific for targets, with GAPDH for normalization. All qPCR reactions were performed in technical triplicate using the StepOnePlus system (ABI). Primers for qPCR were previously described [42, 43].

### Transfection of oligonucleotides

MiR-4732-3p microRNA mimic or non-targeting (NT) microRNA mimic #1 (Dharmacon) were used for microRNA overexpression. For microRNA inhibition, miR-4732-3p antisense 2’-O-Methyl oligonucleotide (AMO) or non-targeting (NT) antisense 2’-O-Methyl oligonucleotide (Dharmacon) were used. For mRNA knockdown, SMAD2 and SMAD4 SMARTpool siGENOME siRNAs (Dharmacon) or Allstar negative control siRNA (Qiagen) were used.

### Plasmid constructs

For luciferase reporter constructs, a portion of the 3' untranslated region (UTR) of SMAD2 and SMAD4 flanking the predicted miR-4732-3p binding site was amplified and cloned into the XhoI and NotI sites downstream of Renilla luciferase in the psiCheck-2 vector (Promega). The 3’UTR mutated reporters were constructed using the QuikChange II Site-Directed Mutagenesis kit (Stratagene, CA) to mutate the predicted targets of the miR-4732-3p seed sequence. Reporter construct PCR and mutagenesis primers are listed (Additional file 8: Table S7). The CAGA-luc SMAD-responsive Firefly construct was kindly provided by the Dr. Gerard Blobe lab. An empty control Renilla construct was obtained from Promega. The SMAD4 cDNA overexpression construct was obtained from Origene, and the SMAD2 cDNA (HsCD00001922) overexpression construct [65] was obtained from DNASU.

### Data analysis

All statistical analyses were performed using the GraphPad Prism4 software package, and represent unpaired Student t-tests. All graphs were drawn with either GraphPad Prism4 or Microsoft Excel 2014 software packages.

### Availability of supporting data

All additional sequencing data have been deposited in the GEO (Gene Expression Omnibus) data repository with the GEO series accession number GSE63703.

## Additional files

Additional file 1: Figure S1. Confirmation of erythrocyte cell sorting and long RNA read distribution. A) Distribution of cell fluorescence for before sorting (presort), and erythrocyte (CD71-) and reticulocyte (CD71+) populations after sorting in one sample. Cells were stained with the CD71-PE antibody and fluorescence was recorded using flow cytometry. B) Gene body coverage plot from transcription start site (TSS, 5’) to transcription end site (TES, 3’) for listed samples. **Figure S2.** Sequences and folding structures of two predicted putative microRNAs. Precursor coordinates of the predicted microRNA precursors are listed. The most prevalent read sequences for the putative mature microRNA and star sequence from one representative sample are boxed in red and purple, respectively. **Figure S3.** GSEA analysis of enrichment of the predicted target RBC mRNAs vs. PBMC mRNAs for the top six expressed erythrocyte microRNAs. Potential binding sites in mRNA 3’UTRs were identified using Targetscan (release 7.0) [11]. Numbers of predicted target mRNAs for each microRNA are shown. Note that let-7f and let-7a have the exact same seed sequence, so their results are the same. **Figure S4.** RT-PCR validation of lncRNA spanning miR-4732 precursor. A total of 500 ng of erythrocyte RNA from one individual was reverse-transcribed using methods previously described, except with the use of random hexamers for priming, instead of oligo dTs. PCR-amplified lncRNA region is shown. GAPDH is expressed in RBC samples and was used as a positive control. RT reactions without the use of reverse transcriptase (−) were used as a negative control. **Figure S5.** Regulation of miR-486-5p and miR-221 during erythroid differentiation. Expression during CD34+ erythroid differentiation using progenitors from three different individuals. Differentiation is listed from day 6 to day 16. U6 snRNA was used as a loading control, with fold difference set relative to day 6. **Figure S6.** Conservation of miR-451a, miR-144-3p, and miR-144-5p in the listed species. Primates are listed adjacent to white box, non-primates listed adjacent to black box. Seed sequence is boxed, and base mismatches are highlighted in red. (PDF 2445 kb) Additional file 2: Table S1. Summary of all expressed long RNAs. (XLSX 2170 kb) Additional file 3: Table S2. GATHER gene ontology categorization of top 500 expressed genes. (XLSX 70 kb) Additional file 4: Table S3. Summary of known microRNAs. Number of sequence reads was determined with either counts from known microRNA sequence search (Seq search) or miRDeep output, using the highest number of reads for that sequence. Listed genomic locations for mature microRNA sequences are relative to RefSeq annotated transcripts in UCSC genome browser (hg38). For mature microRNA sequences spanning both introns and coding region, or UTR or coding of alternative transcripts, sequence is listed in coding region. Conservation to human (H), chimp (C), rhesus monkey (R), dog (D), mouse (M), and zebrafish (Z) are listed. Conservation of mature microRNA sequence indicates exact sequence alignment in listed species, allowing for up to one base mismatch outside of the microRNA seed sequence (nucleotides 2–8), and no mismatches within the seed sequence. (XLSX 45 kb) Additional file 5: Table S4. Summary of putative microRNAs. Genomic location and conservation were determined with methods used for Additional file 4: Table S3. (XLSX 17 kb) Additional file 6: Table S5. Long RNA transcripts loci spanning pre-microRNA regions. Loci are listed if 20 or greater long RNA-seq reads max per sample spanned the indicated microRNA precursor region. Rank (in average long RNA reads), chromosome and coordinates of precursor, precursor ID, and maximum number of reads spanning precursors are listed for each erythrocyte long RNA-seq sample. MicroRNAs are listed as expressed if any microRNA mapping to the indicated precursor region were present in 100 or greater reads for all short RNA-seq samples. (XLSX 10 kb) Additional file 7: Table S6. List of predicted targets for miR-4732-3p in Targetscan. (XLSX 60 kb) Additional file 8: Table S7. Primers used in this manuscript. (XLSX 10 kb)

## Abbreviations

CPM: Counts per million
GATHER: Gene annotation tool to help explain relationships
GSEA: Gene set enrichment analysis
lncRNA: Long noncoding RNA
RBC: Red blood cells

## References

[1] Zhao G, Yu D, Weiss MJ (2010). MicroRNAs in erythropoiesis. Curr Opin Hematol.

[2] Hamilton AJ (2010). MicroRNA in erythrocytes. Biochem Soc Trans.

[3] O’Brien CA, Harley JB (1990). A subset of hY RNAs is associated with erythrocyte Ro ribonucleoproteins. EMBO J.

[4] Chen SY, Wang Y, Telen MJ, Chi JT (2008). The genomic analysis of erythrocyte microRNA expression in sickle cell diseases. PLoS One.

[5] Rathjen T, Nicol C, McConkey G, Dalmay T (2006). Analysis of short RNAs in the malaria parasite and its red blood cell host. FEBS Lett.

[6] LaMonte G, Philip N, Reardon J, Lacsina JR, Majoros W, Chapman L, Thornburg CD, Telen MJ, Ohler U, Nicchitta CV (2012). Translocation of sickle cell erythrocyte microRNAs into Plasmodium falciparum inhibits parasite translation and contributes to malaria resistance. Cell Host Microbe.

[7] Sangokoya C, Telen MJ, Chi JT (2010). microRNA miR-144 modulates oxidative stress tolerance and associates with anemia severity in sickle cell disease. Blood.

[8] Dore LC, Amigo JD, Dos Santos CO, Zhang Z, Gai X, Tobias JW, Yu D, Klein AM, Dorman C, Wu W (2008). A GATA-1-regulated microRNA locus essential for erythropoiesis. Proc Natl Acad Sci U S A.

[9] Kannan M, Atreya C (2010). Differential profiling of human red blood cells during storage for 52 selected microRNAs. Transfusion.

[10] Azzouzi I, Moest H, Wollscheid B, Schmugge M, Eekels JJ, Speer O (2015). Deep Sequencing and Proteomic Analysis of the MicroRNA-Induced Silencing Complex in Human Red Blood Cells. Exp Hematol.

[11] Lewis BP, Burge CB, Bartel DP (2005). Conserved seed pairing, often flanked by adenosines, indicates that thousands of human genes are microRNA targets. Cell.

[12] He W, Dorn DC, Erdjument-Bromage H, Tempst P, Moore MA, Massague J (2006). Hematopoiesis controlled by distinct TIF1gamma and Smad4 branches of the TGFbeta pathway. Cell.

[13] Sangokoya C, LaMonte G, Chi JT (2010). Isolation and characterization of microRNAs of human mature erythrocytes. Methods Mol Biol.

[14] Wong JJ, Ritchie W, Ebner OA, Selbach M, Wong JW, Huang Y, Gao D, Pinello N, Gonzalez M, Baidya K (2013). Orchestrated intron retention regulates normal granulocyte differentiation. Cell.

[15] Pimentel H, Parra M, Gee S, Ghanem D, An X, Li J, Mohandas N, Pachter L, Conboy JG (2014). A dynamic alternative splicing program regulates gene expression during terminal erythropoiesis. Nucleic Acids Res.

[16] Fried VA, Smith HT, Hildebrandt E, Weiner K (1987). Ubiquitin has intrinsic proteolytic activity: implications for cellular regulation. Proc Natl Acad Sci U S A.

[17] Thom CS, Traxler EA, Khandros E, Nickas JM, Zhou OY, Lazarus JE, Silva AP, Prabhu D, Yao Y, Aribeana C (2014). Trim58 degrades Dynein and regulates terminal erythropoiesis. Dev Cell.

[18] Song H, Luo J, Luo W, Weng J, Wang Z, Li B, Li D, Liu M (2008). Inactivation of G-protein-coupled receptor 48 (Gpr48/Lgr4) impairs definitive erythropoiesis at midgestation through down-regulation of the ATF4 signaling pathway. J Biol Chem.

[19] Cokic VP, Smith RD, Biancotto A, Noguchi CT, Puri RK, Schechter AN (2013). Globin gene expression in correlation with G protein-related genes during erythroid differentiation. BMC Genomics.

[20] Zhang J, Loyd MR, Randall MS, Waddell MB, Kriwacki RW, Ney PA (2012). A short linear motif in BNIP3L (NIX) mediates mitochondrial clearance in reticulocytes. Autophagy.

[21] Shaw GC, Cope JJ, Li L, Corson K, Hersey C, Ackermann GE, Gwynn B, Lambert AJ, Wingert RA, Traver D (2006). Mitoferrin is essential for erythroid iron assimilation. Nature.

[22] Ponka P, Beaumont C, Richardson DR (1998). Function and regulation of transferrin and ferritin. Semin Hematol.

[23] Conboy J, Kan YW, Shohet SB, Mohandas N (1986). Molecular cloning of protein 4.1, a major structural element of the human erythrocyte membrane skeleton. Proc Natl Acad Sci U S A.

[24] Kohno K, Narimatsu H, Shiono Y, Suzuki I, Kato Y, Fukao A, Kubota I, Ueno Y, Kayama T, Kato T (2014). Management of erythropoiesis: cross-sectional study of the relationships between erythropoiesis and nutrition, physical features, and adiponectin in 3519 Japanese people. Eur J Haematol.

[25] Friedlander MR, Mackowiak SD, Li N, Chen W, Rajewsky N (2012). miRDeep2 accurately identifies known and hundreds of novel microRNA genes in seven animal clades. Nucleic Acids Res.

[26] Swaminathan S, Hu X, Zheng X, Kriga Y, Shetty J, Zhao Y, Stephens R, Tran B, Baseler MW, Yang J (2013). Interleukin-27 treated human macrophages induce the expression of novel microRNAs which may mediate anti-viral properties. Biochem Biophys Res Commun.

[27] Sharbati S, Friedlander MR, Sharbati J, Hoeke L, Chen W, Keller A, Stahler PF, Rajewsky N, Einspanier R (2010). Deciphering the porcine intestinal microRNA transcriptome. BMC Genomics.

[28] Jima DD, Zhang J, Jacobs C, Richards KL, Dunphy CH, Choi WW, Au WY, Srivastava G, Czader MB, Rizzieri DA (2010). Deep sequencing of the small RNA transcriptome of normal and malignant human B cells identifies hundreds of novel microRNAs. Blood.

[29] Wang LS, Li L, Li L, Chu S, Shiang KD, Li M, Sun HY, Xu J, Xiao FJ, Sun G (2014). MicroRNA-486 regulates normal erythropoiesis and enhances growth and modulates drug response in CML progenitors. Blood.

[30] Shaham L, Vendramini E, Ge Y, Goren Y, Birger Y, Tijssen MR, McNulty M, Geron I, Schwartzman O, Goldberg L (2014). MicroRNA-486-5p is an erythroid oncomiR of the myeloid leukemias of Down syndrome. Blood.

[31] Yu D, dos Santos CO, Zhao G, Jiang J, Amigo JD, Khandros E, Dore LC, Yao Y, D’Souza J, Zhang Z (2010). miR-451 protects against erythroid oxidant stress by repressing 14-3-3zeta. Genes Dev.

[32] Fu YF, Du TT, Dong M, Zhu KY, Jing CB, Zhang Y, Wang L, Fan HB, Chen Y, Jin Y (2009). Mir-144 selectively regulates embryonic alpha-hemoglobin synthesis during primitive erythropoiesis. Blood.

[33] Patrick DM, Zhang CC, Tao Y, Yao H, Qi X, Schwartz RJ, Jun-Shen Huang L, Olson EN (2010). Defective erythroid differentiation in miR-451 mutant mice mediated by 14-3-3zeta. Genes Dev.

[34] Byon JC, Padilla SM, Papaynnopoulou T (2014). Deletion of Dicer in late erythroid cells results in impaired stress erythropoiesis in mice. Exp Hematol.

[35] Noh SJ, Miller SH, Lee YT, Goh SH, Marincola FM, Stroncek DF, Reed C, Wang E, Miller JL (2009). Let-7 microRNAs are developmentally regulated in circulating human erythroid cells. J Transl Med.

[36] Teruel-Montoya R, Kong X, Abraham S, Ma L, Kunapuli SP, Holinstat M, Shaw CA, McKenzie SE, Edelstein LC, Bray PF (2014). MicroRNA expression differences in human hematopoietic cell lineages enable regulated transgene expression. PLoS One.

[37] Kirschner MB, Kao SC, Edelman JJ, Armstrong NJ, Vallely MP, van Zandwijk N, Reid G (2011). Haemolysis during sample preparation alters microRNA content of plasma. PLoS One.

[38] Pritchard CC, Kroh E, Wood B, Arroyo JD, Dougherty KJ, Miyaji MM, Tait JF, Tewari M (2012). Blood cell origin of circulating microRNAs: a cautionary note for cancer biomarker studies. Cancer Prev Res (Phila).

[39] Bruchova H, Yoon D, Agarwal AM, Mendell J, Prchal JT (2007). Regulated expression of microRNAs in normal and polycythemia vera erythropoiesis. Exp Hematol.

[40] Chang JT, Nevins JR (2006). GATHER: a systems approach to interpreting genomic signatures. Bioinformatics.

[41] Nakao A, Imamura T, Souchelnytskyi S, Kawabata M, Ishisaki A, Oeda E, Tamaki K, Hanai J, Heldin CH, Miyazono K, ten Dijke P (1997). TGF-beta receptor-mediated signalling through Smad2, Smad3 and Smad4. EMBO J.

[42] Chen YG, Wang Z, Ma J, Zhang L, Lu Z (2007). Endofin, a FYVE domain protein, interacts with Smad4 and facilitates transforming growth factor-beta signaling. J Biol Chem.

[43] Dong XM, Yin RH, Yang Y, Feng ZW, Ning HM, Dong L, Zheng WW, Tang LJ, Wang J, Jia YX (2014). GATA-2 inhibits transforming growth factor-beta signaling pathway through interaction with Smad4. Cell Signal.

[44] Harada H, Grant S (2003). Apoptosis regulators. Rev Clin Exp Hematol.

[45] Balsara RD, Ploplis VA (2008). Plasminogen activator inhibitor-1: the double-edged sword in apoptosis. Thromb Haemost.

[46] Ferrandiz N, Caraballo JM, Garcia-Gutierrez L, Devgan V, Rodriguez-Paredes M, Lafita MC, Bretones G, Quintanilla A, Munoz-Alonso MJ, Blanco R (2012). p21 as a transcriptional co-repressor of S-phase and mitotic control genes. PLoS One.

[47] Zermati Y, Fichelson S, Valensi F, Freyssinier JM, Rouyer-Fessard P, Cramer E, Guichard J, Varet B, Hermine O (2000). Transforming growth factor inhibits erythropoiesis by blocking proliferation and accelerating differentiation of erythroid progenitors. Exp Hematol.

[48] Randrianarison-Huetz V, Laurent B, Bardet V, Blobe GC, Huetz F, Dumenil D (2010). Gfi-1B controls human erythroid and megakaryocytic differentiation by regulating TGF-beta signaling at the bipotent erythro-megakaryocytic progenitor stage. Blood.

[49] Choi SJ, Moon JH, Ahn YW, Ahn JH, Kim DU, Han TH (2005). Tsc-22 enhances TGF-beta signaling by associating with Smad4 and induces erythroid cell differentiation. Mol Cell Biochem.

[50] Dussiot M, Maciel TT, Fricot A, Chartier C, Negre O, Veiga J, Grapton D, Paubelle E, Payen E, Beuzard Y (2014). An activin receptor IIA ligand trap corrects ineffective erythropoiesis in beta-thalassemia. Nat Med.

[51] Suragani RN, Cadena SM, Cawley SM, Sako D, Mitchell D, Li R, Davies MV, Alexander MJ, Devine M, Loveday KS (2014). Transforming growth factor-beta superfamily ligand trap ACE-536 corrects anemia by promoting late-stage erythropoiesis. Nat Med.

[52] Dennler S, Itoh S, Vivien D, ten Dijke P, Huet S, Gauthier JM (1998). Direct binding of Smad3 and Smad4 to critical TGF beta-inducible elements in the promoter of human plasminogen activator inhibitor-type 1 gene. EMBO J.

[53] Beelman CA, Parker R (1995). Degradation of mRNA in eukaryotes. Cell.

[54] He L, Hannon GJ (2004). MicroRNAs: small RNAs with a big role in gene regulation. Nat Rev Genet.

[55] Lin S, Cheung WK, Chen S, Lu G, Wang Z, Xie D, Li K, Lin MC, Kung HF (2010). Computational identification and characterization of primate-specific microRNAs in human genome. Comput Biol Chem.

[56] Lopez JP, Lim R, Cruceanu C, Crapper L, Fasano C, Labonte B, Maussion G, Yang JP, Yerko V, Vigneault E (2014). miR-1202 is a primate-specific and brain-enriched microRNA involved in major depression and antidepressant treatment. Nat Med.

[57] Pishesha N, Thiru P, Shi J, Eng JC, Sankaran VG, Lodish HF (2014). Transcriptional divergence and conservation of human and mouse erythropoiesis. Proc Natl Acad Sci U S A.

[58] Rasmussen KD, Simmini S, Abreu-Goodger C, Bartonicek N, Di Giacomo M, Bilbao-Cortes D, Horos R, Von Lindern M, Enright AJ, O’Carroll D (2010). The miR-144/451 locus is required for erythroid homeostasis. J Exp Med.

[59] Attie KM, Allison MJ, McClure T, Boyd IE, Wilson DM, Pearsall AE, Sherman ML (2014). A phase 1 study of ACE-536, a regulator of erythroid differentiation, in healthy volunteers. Am J Hematol.

[60] Kersey PJ, Staines DM, Lawson D, Kulesha E, Derwent P, Humphrey JC, Hughes DS, Keenan S, Kerhornou A, Koscielny G (2012). Ensembl Genomes: an integrative resource for genome-scale data from non-vertebrate species. Nucleic Acids Res.

[61] Dobin A, Davis CA, Schlesinger F, Drenkow J, Zaleski C, Jha S, Batut P, Chaisson M, Gingeras TR (2013). STAR: ultrafast universal RNA-seq aligner. Bioinformatics.

[62] Wang L, Wang S, Li W (2012). RSeQC: quality control of RNA-seq experiments. Bioinformatics.

[63] Trapnell C, Hendrickson DG, Sauvageau M, Goff L, Rinn JL, Pachter L (2013). Differential analysis of gene regulation at transcript resolution with RNA-seq. Nat Biotechnol.

[64] Subramanian A, Tamayo P, Mootha VK, Mukherjee S, Ebert BL, Gillette MA, Paulovich A, Pomeroy SL, Golub TR, Lander ES, Mesirov JP (2005). Gene set enrichment analysis: a knowledge-based approach for interpreting genome-wide expression profiles. Proc Natl Acad Sci U S A.

[65] Witt AE, Hines LM, Collins NL, Hu Y, Gunawardane RN, Moreira D, Raphael J, Jepson D, Koundinya M, Rolfs A (2006). Functional proteomics approach to investigate the biological activities of cDNAs implicated in breast cancer. J Proteome Res.

