# Supplementary material for: A comprehensive joint analysis of the long and short RNA transcriptomes of human erythrocytes
Source: BMC Genomics. 2015 Nov 16;16:952. doi: 10.1186/s12864-015-2156-2 (PMC4647483; doi:10.1186/s12864-015-2156-2)
Supplement: Additional file 1: Figure S1. — Confirmation of erythrocyte cell sorting and long RNA read distribution. A) Distribution of cell fluorescence for before sorting (presort), and erythrocyte (CD71-) and reticulocyte (CD71+) populations after sorting in one sample. Cells were stained with the CD71-PE antibody and fluorescence was recorded using flow cytometry. B) Gene body coverage plot from transcription start site (TSS, 5’) to transcription end site (TES, 3’) for listed samples. Figure S2. Sequences and folding structures of two predicted putative microRNAs. Precursor coordinates of the predicted microRNA precursors are listed. The most prevalent read sequences for the putative mature microRNA and star sequence from one representative sample are boxed in red and purple, respectively. Figure S3. GSEA analysis of enrichment of the predicted target RBC mRNAs vs. PBMC mRNAs for the top six expressed erythrocyte microRNAs. Potential binding sites in mRNA 3’UTRs were identified using Targetscan (release 7.0) [11]. Numbers of predicted target mRNAs for each microRNA are shown. Note that let-7f and let-7a have the exact same seed sequence, so their results are the same. Figure S4. RT-PCR validation of lncRNA spanning miR-4732 precursor. A total of 500 ng of erythrocyte RNA from one individual was reverse-transcribed using methods previously described, except with the use of random hexamers for priming, instead of oligo dTs. PCR-amplified lncRNA region is shown. GAPDH is expressed in RBC samples and was used as a positive control. RT reactions without the use of reverse transcriptase (−) were used as a negative control. Figure S5. Regulation of miR-486-5p and miR-221 during erythroid differentiation. Expression during CD34+ erythroid differentiation using progenitors from three different individuals. Differentiation is listed from day 6 to day 16. U6 snRNA was used as a loading control, with fold difference set relative to day 6. Figure S6. Conservation of miR-451a, miR-144-3p, and miR-144-5p in the listed s [file 12864_2015_2156_MOESM1_ESM.pdf]

FIGURE S1

A

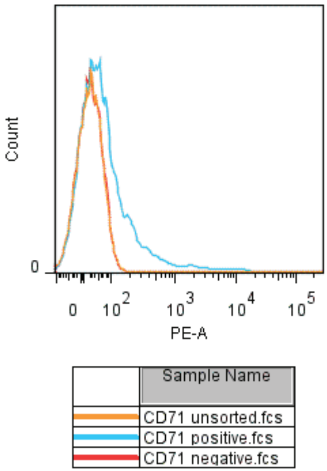

Count
0
0
10^2
10^3
10^4
10^5
PE-A
Sample Name
CD71 unsorted.fcs
CD71 positive.fcs
CD71 negative.fcs

B

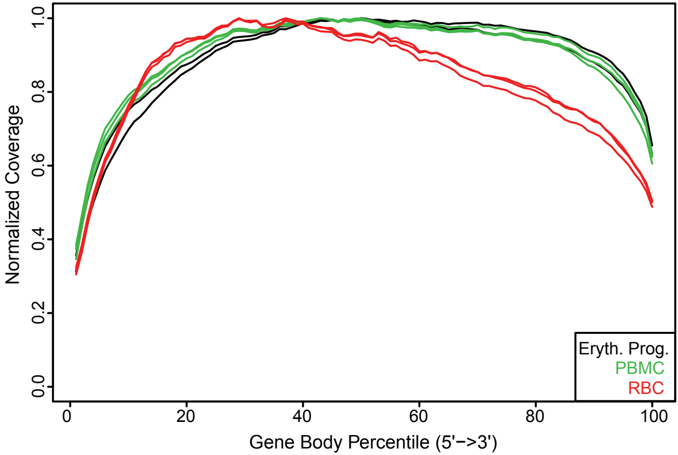

Normalized Coverage
0.0
0.2
0.4
0.6
0.8
1.0
0
20
40
60
80
100
Gene Body Percentile (5'−>3')
Eryth. Prog.
PBMC
RBC

FIGURE S2

chr22:20012651..20012708:-

Reads	Star Sequence	Mature MicroRNA Sequence	Reads
		CACTGCAACCTCTGCCTCCGG	86
3	CTGAGAGGCAGAGGTTGCAG	CACTGCAACCTCTGCCTCCGGTA	87
4	CTGAGAGGCAGAGGTTGCAGT	CACTGCAACCTCTGCCTCCGGA	123
4	CTGAGAGGCAGAGGTTGCAGTG	CACTGCAACCTCTGCCTCCGGT	304

ttgaacCTGAGAGGCAGAGGTTGCAGTGGcacgatctcaggtCACTGCAACCTCTGCCTCCGGTattcaa

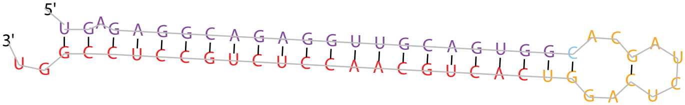

Relative Genomic Location	miRDeep score	Total Reads per sample				
		Sample 1	Sample 2	Sample 3	Sample 4	Sample5
Intron, TANGO2	660	0	1269	0	0	403

chr7:1538188..1538248:-

Reads	Star Sequence	Mature MicroRNA Sequence	Reads
		CTGGACACTGACCAGGACCCCCT	16
		CTGGACACTGACCAGGACCCCT	30
1	GCCCTGGCCTGGATCCATGCTG	CTGGACACTGACCAGGACCCCC	2

ggggGCCCTGGCCTGGATCCATGCTGggcagaagcagCTGGACACTGACCAGGACCCCCcag

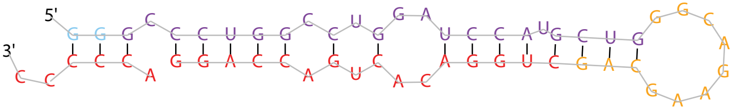

Relative Genomic Location	miRDeep score	Total Reads per sample				
		Sample 1	Sample 2	Sample 3	Sample 4	Sample5
Intron, EGFL1	1.6	0	112	17	0	49

FIGURE S3

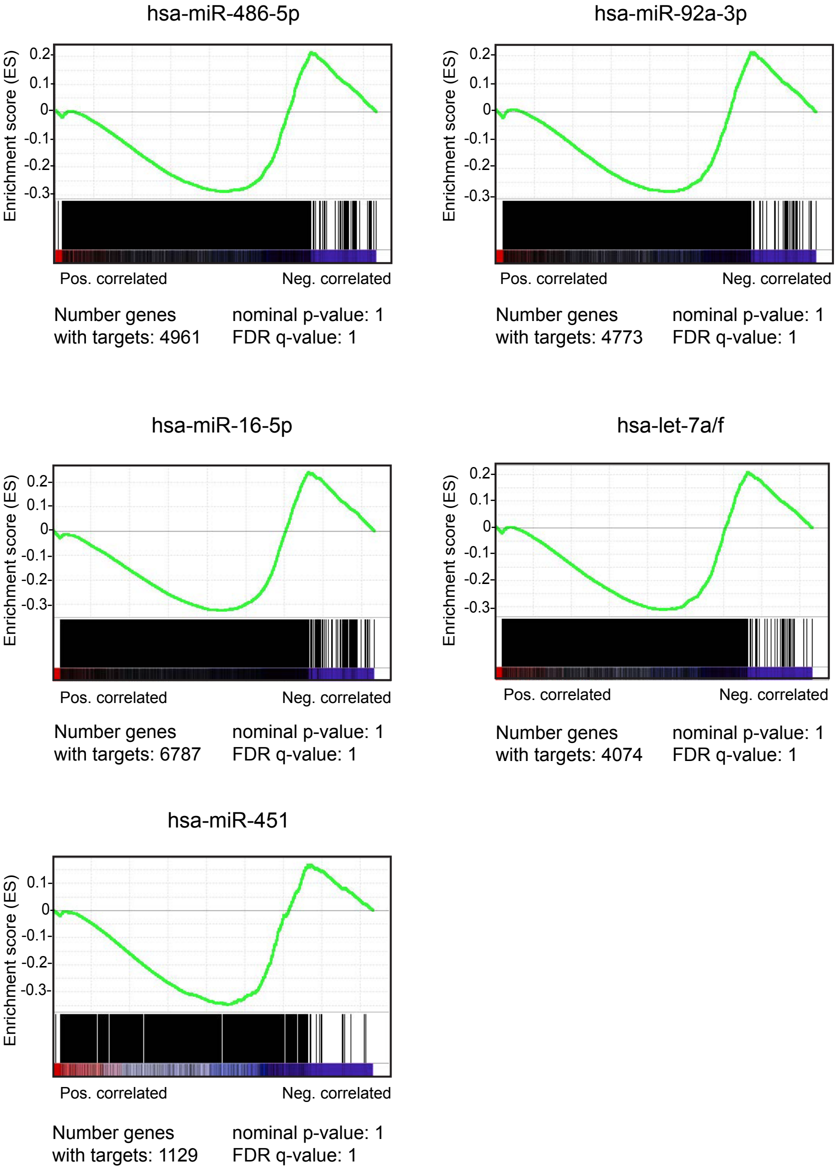
hsa-miR-486-5p
Enrichment score (ES)
0.2
0.1
0
-0.1
-0.2
-0.3
Pos. correlated
Neg. correlated
Number genes with targets: 4961
nominal p-value: 1
FDR q-value: 1
hsa-miR-92a-3p
Enrichment score (ES)
0.2
0.1
0
-0.1
-0.2
-0.3
Pos. correlated
Neg. correlated
Number genes with targets: 4773
nominal p-value: 1
FDR q-value: 1
hsa-miR-16-5p
Enrichment score (ES)
0.2
0.1
0
-0.1
-0.2
-0.3
Pos. correlated
Neg. correlated
Number genes with targets: 6787
nominal p-value: 1
FDR q-value: 1
hsa-let-7a/f
Enrichment score (ES)
0.2
0.1
0
-0.1
-0.2
-0.3
Pos. correlated
Neg. correlated
Number genes with targets: 4074
nominal p-value: 1
FDR q-value: 1
hsa-miR-451
Enrichment score (ES)
0.1
0
-0.1
-0.2
-0.3
Pos. correlated
Neg. correlated
Number genes with targets: 1129
nominal p-value: 1
FDR q-value: 1

FIGURE S4

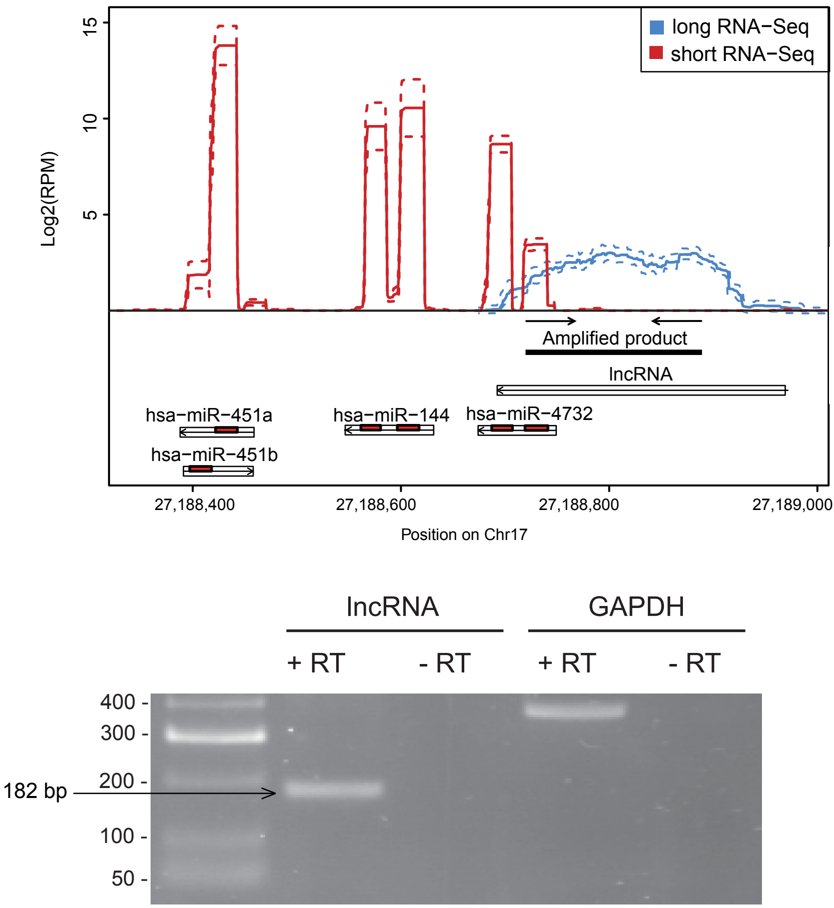

long RNA−Seq
short RNA−Seq
Log2(RPM)
15
10
5
Amplified product
lncRNA
hsa−miR−451a
hsa−miR−451b
hsa−miR−144
hsa−miR−4732
27,188,400
27,188,600
27,188,800
27,189,000
Position on Chr17
lncRNA
GAPDH
+ RT
- RT
+ RT
- RT
400 -
300 -
200 -
100 -
50 -
182 bp

FIGURE S5

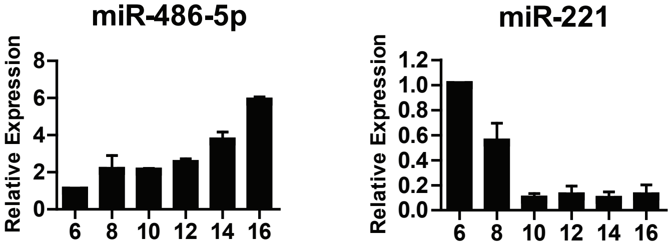

miR-486-5p
Relative Expression
8
6
4
2
0
6
8
10
12
14
16
miR-221
Relative Expression
1.2
1.0
0.8
0.6
0.4
0.2
0.0
6
8
10
12
14
16

FIGURE S6

miR-451a

Human	AAACCGTTACCATTACTGAGTT
Chimpanzee	AAACCGTTACCATTACTGAGTT
Gorilla	AAACCGTTACCATTACTGAGTT
Orangutan	AAACCGTTACCATTACTGAGTT
Macaque	AAACCGTTACCATTACTGAGTT
Mouse Lemur	AAACCGTTACCATTACTGAGTT
Rat	AAACCGTTACCATTACTGAGTT
Mouse	AAACCGTTACCATTACTGAGTT
Rabbit	AAACCGTTACCATTACTGAGTT
Cow	AAACCGTTACCATTACTGAGTT
Dog	AAACCGTTACCATTACTGAGTT
Cat	AAACCGTTACCATTACTGAGTT
Horse	AAACCGTTACCATTACTGTGTT
Chicken	AAACCGTTACCATTACTGAGTT

miR-144-3p

Human	TACAGTATAGATGATGTACT
Chimpanzee	TACAGTATAGATGATGTACT
Gorilla	TACAGTATAGATGATGTACT
Orangutan	TACAGTATAGATGATGTACT
Macaque	TACAGTATAGATGATGTACT
Mouse Lemur	TACAGTATAGATGATGTACT
Rat	TACAGTATAGATGATGTACT
Mouse	TACAGTATAGATGATGTACT
Rabbit	TACAGTATAGATGATGTACT
Cow	TACAGTATAGATGATGTACT
Dog	TACAGTATAGATGATGTACT
Cat	TACAGTATAGATGATGTACT
Horse	TACAGTATAGATGATGTACT
Chicken	TACAGTATAGATGATGTACT

miR-144-5p

Human	GGATATCATCATATACTGTAAG
Chimpanzee	GGATATCATCATATACTGTAAG
Gorilla	GGATATCATCATATACTGTAAG
Orangutan	GGATATCATCATATACTGTAAG
Macaque	GGATATCATCATATACTGTAAG
Mouse Lemur	GGATATCATCATATACTGTAAG
Rat	GGATATCATCATATACTGTAAG
Mouse	GGATATCATCATATACTGTAAG
Rabbit	GGATATCATCATATACTGTAAG
Cow	GGATATCATCATATACTGTAAG
Dog	GGATATCATCATATACTGTAAG
Cat	GGATATCATCATATACTGTAAG
Horse	GGATATCATCATATACTGTAAG
Chicken	GGATATCATCATATACTGTAAG
